# Multifunctional Hypercrosslinked Porous Organic Polymers Based on Tetraphenylethene and Triphenylamine Derivatives for High-Performance Dye Adsorption and Supercapacitor

**DOI:** 10.3390/polym12102426

**Published:** 2020-10-21

**Authors:** Mohamed Gamal Mohamed, Ahmed. F. M. EL-Mahdy, Tso-Shiuan Meng, Maha Mohamed Samy, Shiao-Wei Kuo

**Affiliations:** 1 Department of Materials and Optoelectronic Science, Center of Crystal Research, National Sun Yat-Sen University, Kaohsiung 80424, Taiwan; mgamal.eldin34@gmail.com (M.G.M.); ahmed1932005@gmail.com (A.F.M.E.-M.); s199483226@gmail.com (T.-S.M.); maha35430@gmail.com (M.M.S.); 2 Chemistry Department, Faculty of Science, Assiut University, Assiut 71516, Egypt; 3 Department of Medicinal and Applied Chemistry, Kaohsiung Medical University, Kaohsiung 807, Taiwan

**Keywords:** tetraphenylethene, triphenylamine, porous polymers, dyes adsorption, supercapacitor

## Abstract

We successfully prepared two different classes of hypercrosslinked porous organic polymers (HPPs)—the tetraphenylethene (TPE) and (4-(5,6-Diphenyl-1H-Benzimidazol-2-yl)-triphenylamine (DPT) HPPs—through the Friedel−Crafts polymerization of tetraphenylethene and 4-(5,6-diphenyl-1H-benzimidazol-2-yl)-triphenylamine, respectively, with 1,4-bis(chloromethyl)benzene (Ph-2Cl) in the presence of anhydrous FeCl_3_ as a catalyst. Our porous materials exhibited high BET surface areas (up to 1000 m^2^ g^−1^) and good thermal stabilities. According to electrochemical and dyes adsorption applications, the as-prepared DPT-HPP exhibited a high specific capacitance of 110 F g^−1^ at a current density of 0.5 A g^−1^, with an excellent cycling stability of over 2000 times at 10 A g^−1^. In addition, DPT-HPP showed a high adsorption capacity up to 256.40 mg g^−1^ for the removal of RhB dye from water.

## 1. Introduction

Microporous organic polymers (MOPs) have attracted much attention as next-generation materials in the industry and academic areas because of their good thermal stability, low density, low regeneration energy, high pore volume and large BET (Brunauer–Emmett–Teller) surface area, synthetic diversity, and easier preparation [1,2,3,4,5,6,7]. MOPs have been used in many potential applications, such as water treatment, drug delivery, chemical sensing, heterogeneous catalysis, energy storage, hydrogen evolution, nanofiltration, oil scavenging, carbon dioxide reduction, gas separation and gas storage [8,9,10,11,12,13,14,15,16,17]. There are different kinds of MOPs, such as conjugated microporous polymers (CMPs) [15,16,17,18], covalent organic frameworks (COFs) [16,17,18,19,20], polymers of intrinsic microporosity (PIMs) [21,22,23], covalent triazine-based frameworks (CTFs) [24,25,26,27,28], and hypercrosslinked polymers (HCPs) [29,30,31,32].

Among MOPs, HCPs can be easily synthesized through the simple Friedel–Crafts alkylation reaction in the presence of building aromatic monomers and an external crosslinker [33,34,35]. These materials exhibited many unique features, such as large surface areas, excellent chemical and thermal properties, controlled reaction conditions, and easy functionalizations. Moreover, HCPs can be synthesized to afford a framework with a well-defined porous topology by controlling the length of the crosslinker [36,37,38,39,40,41]. The preparation HCPs can be easily synthesized through three methods: (i) knitting rigid aromatic building blocks with an external crosslinker, such as 1,3,5-trichlorotriazine, 1,4-bis(chloromethyl)benzene or formaldehyde dimethyl acetal, (ii) the one-step polycondensation of functional monomers, and (iii) using post-crosslinking polymer precursors [39,42]. At present, HCP materials are still interesting porous polymers for energy storage applications because of their hierarchical porous structures, the extended π-conjugated systems in their frameworks, and their large surface areas [43,44,45]. The enhancement of the electronic conductivity, electroactive surface area and supercapacitor performance of the HCP materials can be achieved through the incorporation of heteroatoms such as sulfur, nitrogen, boron, and phosphorus into their polymeric frameworks, and also by using versatile building blocks for increasing their pore volumes and BET surface areas [46,47]. The preparation of heterocyclic imidazole derivatives has drawn much attention because of their unusual fluorescent, two-photon absorption, and phosphorescent properties. Such materials have been also used as optical materials, electroluminescent materials, three-dimensional optical data storages, and photographic materials [48,49,50,51,52,53,54,55].

Herein, we prepared two types of hypercrosslinked porous organic polymers, TPE-HPP and DPT-HPP, using the Friedel−Crafts polymerization of tetraphenylethene (TPE) and 4-(5,6-diphenyl-1H-benzimidazol- 2-yl)-triphenylamine (DPT), respectively, with 1,4-bis(chloromethyl)benzene (Ph-2Cl) as an external crosslinker in the presence of anhydrous FeCl_3_. The chemical structures of these materials were confirmed by solid-state ^13^C cross-polarization/magic-angle spinning (CP/MAS) nuclear magnetic resonance (NMR) and Fourier-transform infrared (FTIR) spectroscopy. Furthermore, the porous structure, morphology and crystallinity of these two porous materials were also investigated using different instruments, such as SEM, TEM, BET and PXRD measurements. We found that the resulting materials displayed high thermal stabilities and surface areas. Thus, we expect that our new hypercrosslinked polymers TPE-HPP and DPT-HPP could be used in energy storage and dye-uptake applications.

## 2. Experimental Section

### 2.1. Materials

Benzophenone (99%), potassium carbonate (K_2_CO_3_, 99.995%), rhodamine B (RhB, 95%), and 1,4-bis(chloromethyl)benzene (98%) were purchased from Alfa Aesar. Titanium tetrachloride (TiCl_4_, 99.9%), zinc (Zn, 98%), phosphorus trichloride (POCl_3_, 99%), benzil (98%), ammonium acetate (CH_3_COONH_4_, 98%), acetic acid (CH_3_COOH, 99%), and 1,2-dichloroethane (DCE, 99.8%) were purchased from Alfa Aesar. Triphenylamine (TPA, 98%) and ethyl acetate (EA) were ordered from Acros Organics. Anhydrous magnesium sulfate (MgSO_4_, 99.5%) and iron(III) chloride (FeCl_3_, 99.99%), tetrahydrofuran (THF), acetone, methanol (CH_3_OH), dimethyl sulfoxide (DMSO), and dichloromethane (DCM), were purchased from Showa Corporation (Japan). 4-(Diphenylamino)benzaldehyde (TPA-CHO) was prepared as reported in the previous study [50].

### 2.2. Synthesis of Tetraphenylethylene (TPE)

A mixture of benzophenone (5.00 g, 0.027 mol) and zinc (6.52 g, 0.099 mol) in dry THF (200 mL) was stirred at 0 °C for 30 min. Then, TiCl_4_ (5.43 mL, 0.049 mol) was added slowly over 10 min and then the reaction mixture was refluxed at 80 °C for 24 h. The reaction mixture was cooled to room temperature and quenched with a 10% aqueous solution of K_2_CO_3_, followed by the removing of THF under vacuum. The crude product was extracted with ethylacetate (EA), and the organic EA layer was dried over anhydrous MgSO_4_, filtered and removed using a rotary evaporator. The crude product was then washed with ethanol and filtered to afford a white crystalline solid (yield: 97%). ^1^H-NMR (500 MHz, δ, ppm, CDCl_3_, Figure S1): 7.05–7.15 (m, 20H). ^13^C-NMR (125 MHz, δ, ppm, CDCl_3_, Figure S2): 140.7; 141.0; 131.3; 127.7; 126.4.

### 2.3. Synthesis of 4-(5,6-Diphenyl-1H-Benzimidazol-2-yl)-Triphenylamine (DPT)

A mixture of TPA-CHO (1.21 g, 5.74 mmol), benzil (1.00 g, 4.76 mmol), and ammonium acetate (1.84 g, 23.78 mmol) in glacial acetic acid (10 mL) was refluxed at 90 °C for 12 h under a N_2_ atmosphere. The reaction mixture was then poured into an aqueous solution of sodium hydrogen sulfite (200 mL, 3 wt.%) to afford a yellow powder (yield: 80%). 500 MHz, δ, ppm, DMSO-*d*_6_, (Figure S3): 12.54 (s, 1H), 7.99 (d, J = 8.4 Hz, 2H), 7.32–7.52 (m, 14H), 7.03–7.10 (m, 8H). ^13^C-NMR (125 MHz, δ, ppm, DMSO-*d*_6_, Figure S4): 148.2, 147.8, 146.4, 130.5, 129.3, 127.3, 125.3, 125.2, 124.3, 123.6. *m*/*z* (high-resolution mass spectrometry, HRMS) found 463.2050 (M^+^), Anal. Calc. for C_33_H_25_N_3_: 463.2048 (Figure S5).

### 2.4. Synthesis of TPE-HPP

A mixture of tetraphenylethylene (0.523 g, 1.573 mmol) and 1,4-bis(chloromethyl)benzene (1.652 g, 9.44 mmol) in 1,2-dichloroethane (20 mL) was added to anhydrous FeCl_3_ (1.531 g, 9.44 mmol) under an N_2_ atmosphere. The mixture was refluxed at 70 °C for 48 h. After cooling to room temperature, the insoluble residue was filtered off and successively washed several times with methanol, water, DCM, acetone and THF. The product was gained as a brown powder (yield: 75%).

### 2.5. Synthesis of DPT-HPP

A mixture of 4-(5,6-Diphenyl-1H-benzimidazol-2-yl)-triphenylamine (0.521 g, 1.124 mmol) and 1,4-bis(chloromethyl)benzene (1.181 g, 6.744 mmol) in 1,2-dichloroethane (20 mL) was added to anhydrous FeCl_3_ (1.093 g, 6.744 mmol) under a N_2_ atmosphere. The mixture was refluxed at 70 °C for 48 h. After being cooled to room temperature, the insoluble residue was filtered off and successively washed several times with methanol, water, DCM, and tetrahydrofuran (THF). The product was gained as a dark green powder (yield: 80%).

## 3. Results and Discussion

### 3.1. Synthesis of TPE-HPP and DPT-HPP

**Scheme 1:**
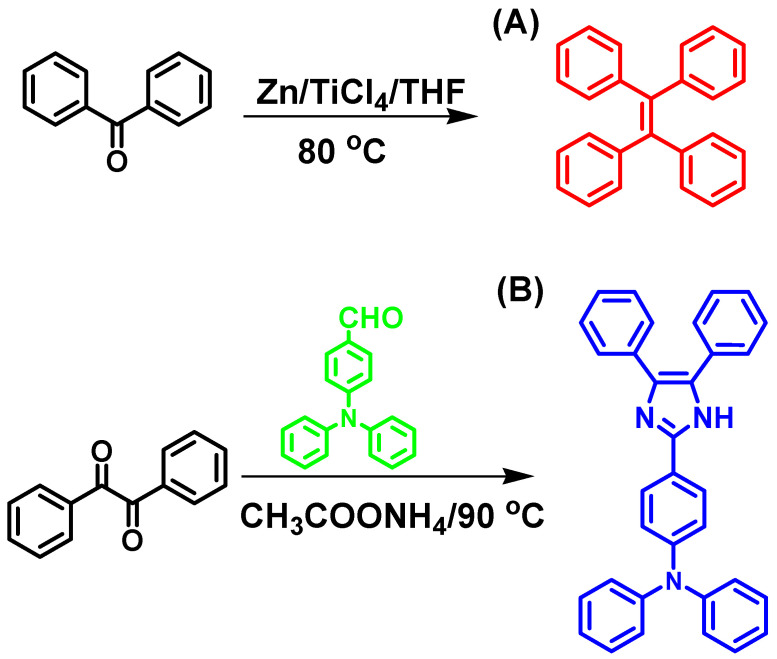
Preparation of (**A**) TPE and (**B**) DPT.

**Scheme 2:**
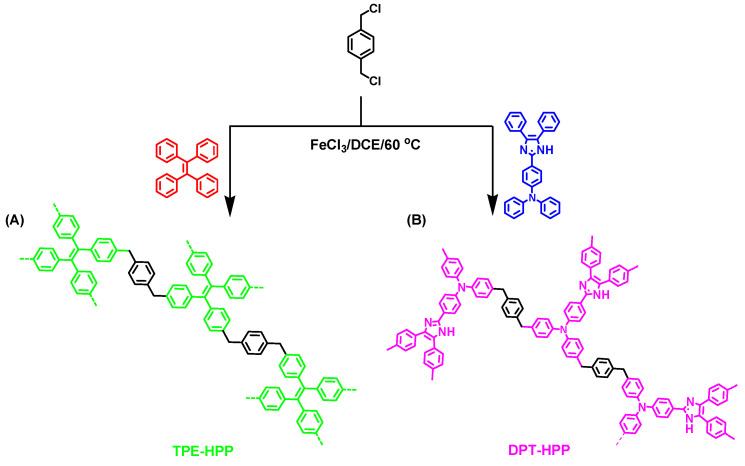
Preparation of (**A**) TPE-HPP and (**B**) DPT-HPP.

Scheme 1**A**,**B** show the synthetic methods for the preparation of TPE and DPT monomers. The TPE monomer was firstly prepared through the reaction of benzophenone with zinc in the presence of TiCl_4_ [56,57], while the DPT monomer was synthesized by the reaction of benzil with 4-(diphenylamino)benzaldehyde (TPA-CHO) and ammonium acetate in acetic acid at 90 °C [50]. Scheme 2 represented the schematic method for preparing the two different HPPs—TPE and DPT HPPs—via a Friedel–Crafts reaction of TPE and DPT with 1,4-bis(chloromethyl)benzene in the presence of anhydrous FeCl_3_ as a catalysis at 60 °C for 24 h. After the reactions were completed, the crude materials were washed several times with MeOH, THF, acetone, DCM, and DMF to remove any excess from the unreacted FeCl_3_ and monomers. In addition, the TPE-HPP and DPT-HPP were insoluble in all organic solvents, such as DMF, acetone, THF, CH_2_Cl_2_ and MeOH.

**Figure 1:**
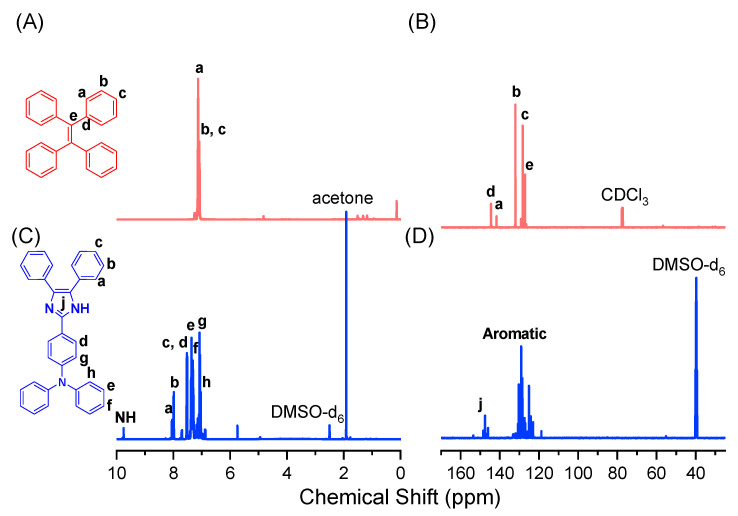
(**A**,**B**) ^1^H-NMR spectra of TPE and DPT. (**C**,**D**) ^13^C-NMR spectra of TPE and DPT.

The chemical molecular structures of TPE and DPT monomers were confirmed by using ^1^H and ^13^C-NMR spectroscopy in CDCl_3_ and DMSO-*d*_6_, respectively (Figure 1). The ^1^H-NMR spectrum (Figure 1**A**) of TPE monomer displayed proton signals centered at 7.14 and 7.04 ppm for the aromatic rings, while the ^1^H-NMR spectrum (Figure 1**B**) of DPT displayed a characteristic proton signal at 9.74 pm for the NH group, in addition to the aromatic proton signals at 8.04–7.04 ppm. Figure 1**C**,**D** presented the ^13^C-NMR analyses of TPE and DPT. The signals of the carbon nuclei of the aromatic rings in the TPE and DPT moieties appeared in the range 144.06–126.56 ppm for TPE and 130.56–122.25 ppm for DPT. Furthermore, the ^13^C-NMR spectrum of DPT (Figure 1**D**) displayed a signal at 147.31 ppm for the C=N carbon nuclei resonance. The molecular weight of the DPT was *m*/*z* 364, which is consistent with its calculated value (*m*/*z* 364.30) (Figure S5). The NMR and high-resolution mass results confirmed the successful synthesis of the TPE and DPT monomers.

**Figure 2:**
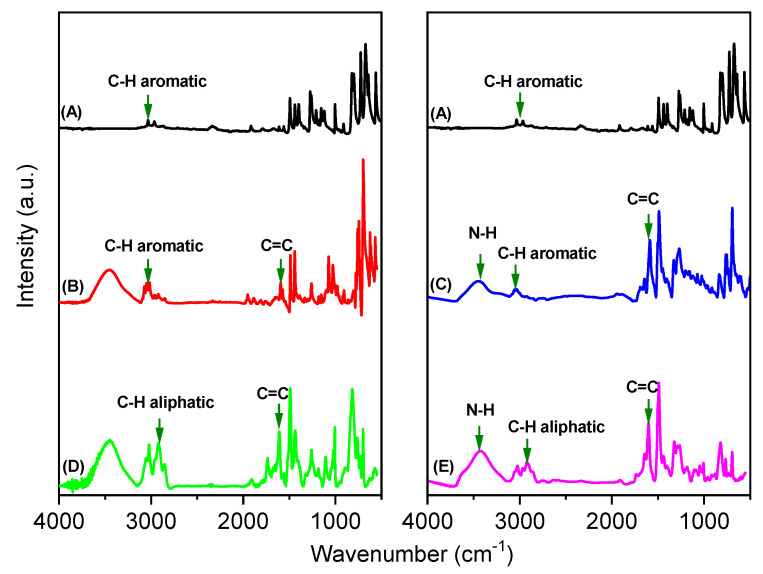
FTIR analyses of (**A**) Ph-2Cl, (**B**) TPE, (**C**) DPT, (**D**) TPE-HPP and (**E**) DPT-HPP.

Figure 2 showed the FTIR profiles of Ph-2Cl, TPE, DPT, TPE-HPP and DPT-HPP, recorded at room temperature. The FTIR spectra of Ph-2Cl, TPE, DPT, TPE-HPP and DPT-HPP showed a characteristic absorption band in the range 3050–3084 cm^–1^ for the stretching C–H aromatic groups, in addition to an absorption band at 1600 cm^–1^ for the C=C bonds. The FTIR spectra of TPE-HPP and DPT-HPP (Figure 2**D**,**E**) featured absorptions signals in the range 2970–2850 cm^−1^ representing their aliphatic C–H units. These results confirmed the successful incorporation of 4-bis(chloromethyl)benzene as an external crosslinker into the polymer matrices. In addition, the absorption bands for the stretching NH groups of the DPT and DPT-HPP appeared at 3435 cm^–1^ (Figure 2**C**,**E**).

**Figure 3:**
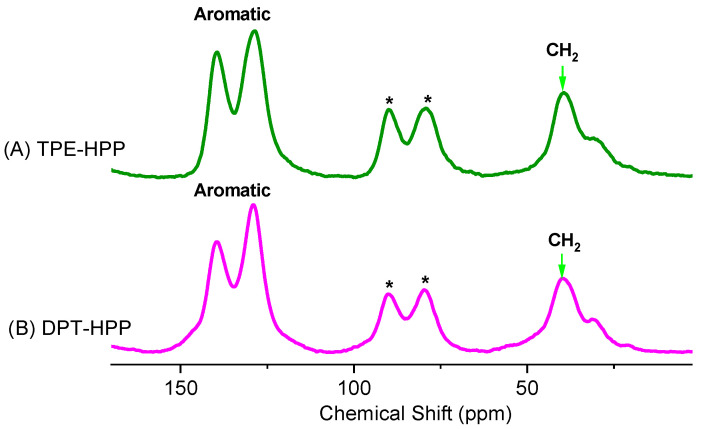
Solid-state ^13^C CP/MAS NMR spectra of (**A**) TPE-HPP and (**B**) DPT-HPP where * is the side band of solid state NMR.

Furthermore, the chemical structures of TPE-HPP and DPT-HPP were checked by using the solid-state ^13^C CP/MAS NMR measurement, as shown in Figure 3. The characteristic signals for the TPE-HPP appeared at 139.67–128.77 and 39.58 ppm, respectively, for its carbon atoms in the aromatic rings and methylene units (Figure 3**A**), while such carbon atoms appeared at 139.49–128.98 ppm and 39.79 ppm, respectively, for the DPT-HPP (Figure 3**B**).

### 3.2. TGA, XRD, BET, SEM, and TEM Analyses for TPE-HPP and DPT-HPP

**Figure 4:**
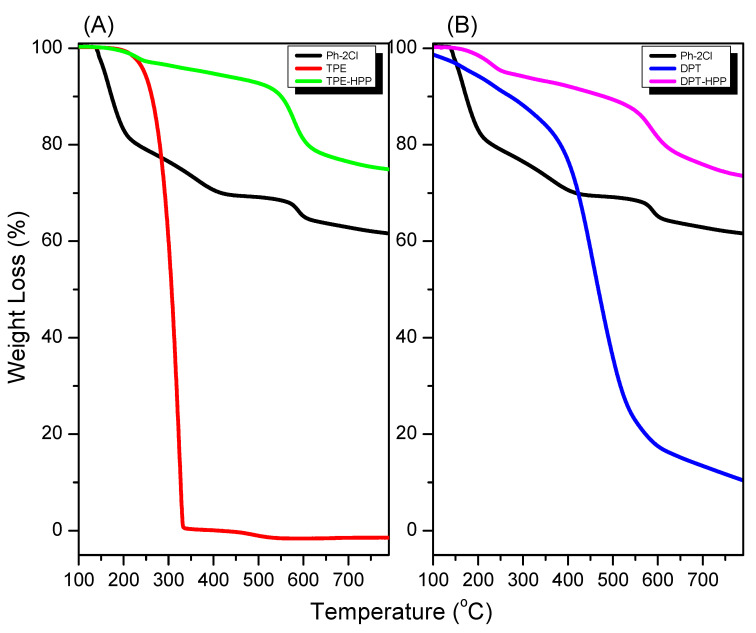
(**A**,**B**) TGA analyses of Ph-2Cl, TPE, DPT, TPE-HPP, and DPT-HPP.

The thermal stabilities of TPE-HPP and DPT-HPP and their corresponding monomers were examined by using TGA analyses under an N_2_ atmosphere, as presented in Figure 4**A**,**B**. The TGA measurements showed that the *T*_d5_, *T*_d10_ and char yield values were 158, 273 °C and 62% for the Ph-2Cl monomer, respectively, while they were 246 °C, 263 and 0%, for the TPE monomer, respectively. The DPT monomer exhibited decomposition temperatures (*T*_d5_ and *T*_d10_) of 186 and 272 °C, respectively, and a char yield value of 11%. After the crosslinking of TPE and DPT with Ph-2Cl through Friedel–Crafts reactions, the values of *T*_d5_ for the resulting TPE-HPP and DPT-HPP were 387 and 263 °C, respectively, and the values of *T*_d10_ were 551 and 481 °C, respectively. In addition, the char yields of TPE-HPP and DPT-HPP were 75% and 74%, respectively. The values of *T*_d5_ and *T*_d10_, and the char yield for TPE-HPP, DPT-HPP and their corresponding monomers, are summarized in Table 1.

**Figure 5:**
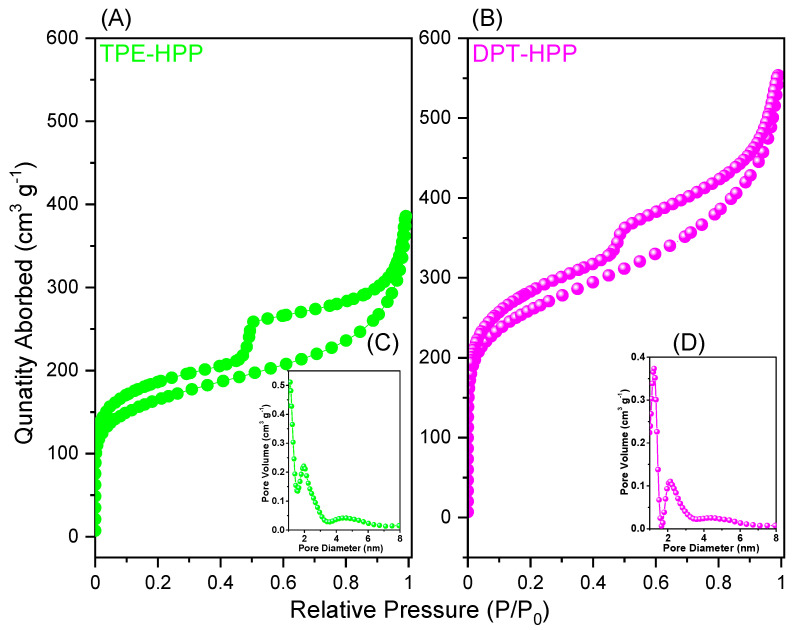
N_2_ adsorption/desorption isotherms of (**A**) TPE-HPP and (**B**) DPT-HPP. Pore size distribution curves of (**C**) TPE-HPP and (**D**) DPT-HPP.

**Figure 6:**
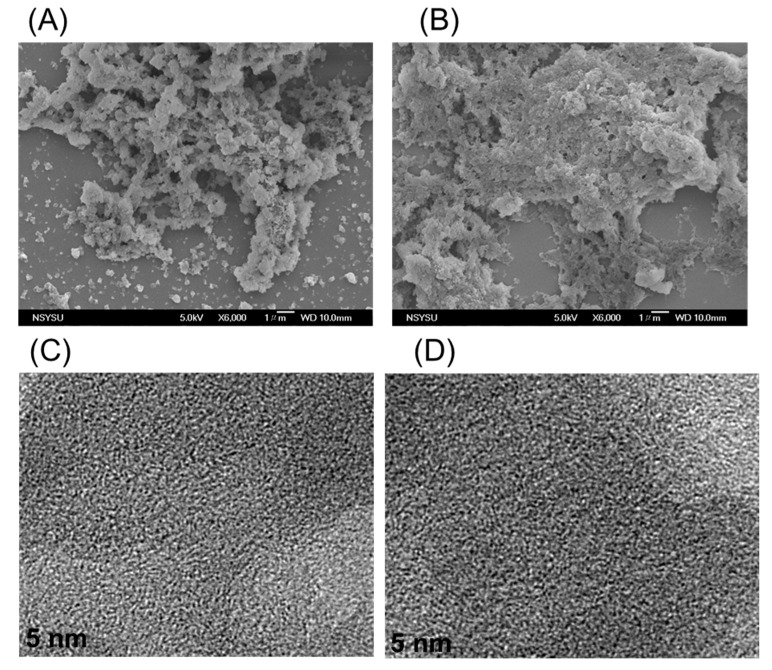
SEM images of (**A**) TPE-HPP and (**B**) DPT-HPP. TEM images of (**C**) TPE-HPP and (**D**) DPT-HPP.

Based on XRD analyses, both TPE-HPP and DPT-HPP were shown to be amorphous materials without crystalline characteristics (Figure S6). Figure 5**A**,**B** show the N_2_ adsorption/desorption and pore size distribution curves of the TPE-HPP and DPT-HPP, respectively, recorded at 1 bar. The results revealed that both TPE-HPP and DPT-HPP featured type II with type IV adsorption isotherms, according to the IUPAC classification. In addition, the BET profiles of TPE-HPP and DPT-HPP displayed that the N_2_ uptake capacities were increased at low-pressure and high-pressure regions, indicating the presence of micro and meso pores in the TPE-HPP and DPT-HPP frameworks. The BET surface area, total pore volume, and pore size diameter of the TPE-HPP were 922 m^2^ g^−1^, 0.86 cm^3^ g^−1^ and 1.10 – 1.98 nm, respectively (Figure 5**C**), while the DPT-HPP revealed a BET surface area, total pore volume and pore size diameter of 1230 m^2^ g^−1^, 1.03 cm^3^ g^−1^ and 1.21–2.15 nm, respectively (Figure 5**D**). Comparing with the reported HCPs, the specific surface area of our DPT-HPP (1230 m^2^ g^−1^) was among the highest values of the other hypercrosslinked microporous polymers; for example, this includes the tetraphenylanthraquinone-based HCP (1980 m^2^ g^−1^), binaphthol-based HCP (1015 m^2^ g^−1^), and a porous organic polymer containing triazine and carbazole moieties (913 m^2^ g^−1^) [20,58,59,60]. Furthermore, the SEM and TEM measurements were performed to confirm the porous properties of the TPE-HPP and DPT-HPP, as displayed in Figure 6. The SEM imaging of TPE-HPP and DPT-HPP showed the existence of interparticulate porosity between the agglomerated primary microgel particles (Figure 6**A**,**B**), while the high-resolution transmission electron imaging (Figure 6**C**,**D**) of the TPE-HPP and DPT-HPP showed the presence of an alternate bright and dark microstructure with a hole shape.

### 3.3. CO_2_ Uptake, Dye Adsorption and Electrochemical Performance

**Figure 7:**
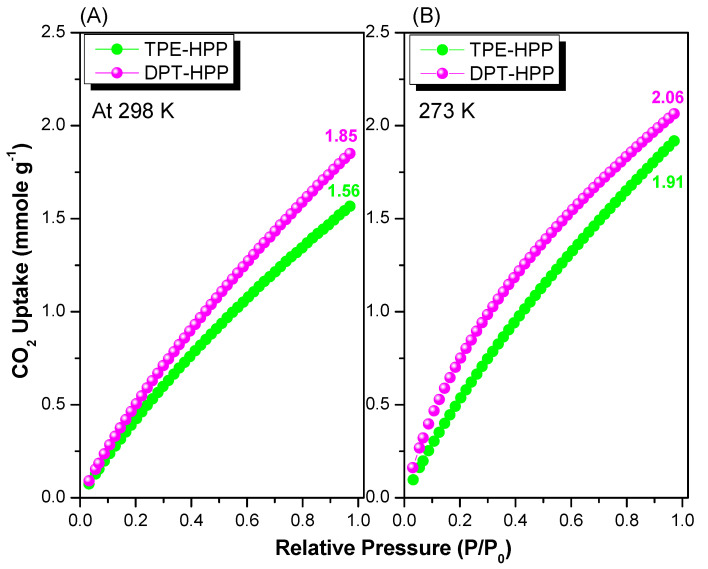
CO_2_ uptake of TPE-HPP and DPT-HPP at (**A**) 298 K and (**B**) 273 K.

Because our porous materials had high surface areas (up to 1000 m^2^ g^−1^), large pore-volumes, and meso and microporous architectures, we expected that our new hypercrosslinked polymers TPE-HPP and DPT-HPP could be used as potential candidates for gas capture, energy storage, and the removal of dyes from the waste-water. CO_2_ isotherm measurements at 298 and 273 K (Figure 7**A**,**B**) were performed to investigate the CO_2_ adsorption capacity of TPE-HPP and DPT-HPP. At 298 K and 1 bar pressure (Figure 7**A**), the CO_2_ adsorption capacity reached 1.65 and 1.85 mmol g^−1^ for the TPE-HPP and DPT-HPP, respectively, while the CO_2_ capture was 1.91 and 2.06 mmol g^−1^ for the TPE-HPP and DPT-HPP, respectively, at 273 K (Figure 7**B**). Both TPE-HPP and DPT-HPP exhibited higher values of CO_2_ uptake than that of the tetraphenylanthraquinone-based HCPs such as An-CPOP-1 (1.30 mmol at 298 K and 1.40 mmol g^−1^ at 273 K) and An-CPOP-2 (1.40 mmol g^−1^ at 298 K and 1.52 mmol g^−1^ at 273 K) [20]. In addition, our materials showed higher CO_2_ capture than silole-based HCPs (PDMTPAS, 1.02 mmol g^−1^ at 298 K), COF-102 (1.56 mmol g^−1^ at 273 K) and C2M1-Al (1.44 mmol g^−1^ at 273 K) [61,62,63]. As shown by the above results, DPT-HPP had an excellent performance for CO_2_ capture compared with the previously reported HCPS materials, which can be attributed to the strong dipole–quadrupole interactions between the DPT-HPP materials and the CO_2_ molecules.

**Figure 8:**
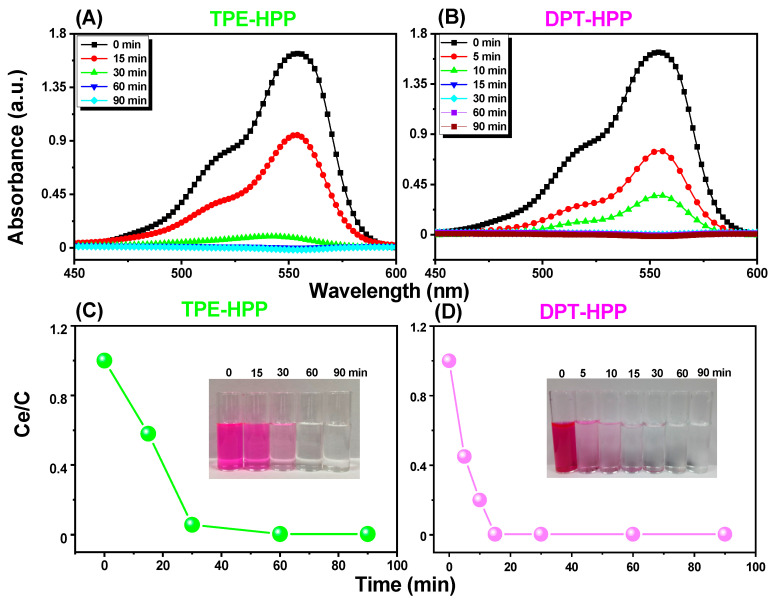
(**A**,**B**) UV–Vis spectra of aqueous RhB recorded at various periods after the addition of TPE-HPP and DPT-HPP (initial concentration of both RhB: 25 mg L^−1^). (**C**,**D**) Adsorption rates of RhB (initial concentration: 25 mg L^−1^) after the addition of TPE-HPP and DPT-HPP at various periods. Insets: Corresponding photographs. *C*_e_ (mg L^−1^) is the equilibrium concentration of RhB at various measured times; *C* (mg L^−1^) is the initial concentration of RhB (0 min).

It has been previously reported that the nitrogen atoms in the organic polymers can strongly interact with dye molecules through non-covalent interactions, such as dipole–dipole, and electrostatic interactions. This interaction ability of nitrogen atoms is due to their basic properties, which resulted from their high charge densities. Therefore, researchers documented that the incorporation of N-functionalized groups, such as azo (N=N) and imine (C=N), into organic polymers can dramatically enhance their adsorption efficiencies towards small organic pollutants in water treatment applications [64]. In addition, the surface area of the adsorbent materials also plays an important role in determining the adsorption efficiency; increasing the surface area massively enhances the adsorption efficiency of the adsorbents [65,66,67,68]. Therefore, we have evaluated the suitability of our hypercrosslinked polymers TPE-HPP and DPT-HPP to be used as adsorbents for water treatment through testing their adsorption capacities to Rhodamine B (RhB) dye. For quantitative evaluation of the adsorptions of TPE-HPP and DPT-HPP polymers, we tracked the RhB adsorption processes by recording the ultraviolet-visible spectra of aqueous dye solutions at 0, 15, 30, 60 and 90 min after the addition of our hypercrosslinked polymers. As shown in Figure 8**A**, after the addition of 5 mg of TPE-HPP hypercrosslinked polymer to aqueous RhB solution, the intensity of the maximum adsorption peak of RhB (λ_max_ = 554 nm) gradually decreased as the time increased from 0 to 30 min, and vanished entirely within 60 min. The insert in Figure 8**C** shows images of RhB/TPE-HPP supernatants at different times—here, the color of the aqueous RhB solution has switched from red-violet to colorless within 60 min. As a result, the TPE-HPP hypercrosslinked polymer revealed high performance for the elimination of RhB dye from water; the removal efficiency of RhB using TPE-HPP reached up to 97% within 60 min (Figure 8**C**). As shown in Figure 8**B**, the intensity of the maximum adsorption peak of RhB gradually decreased from 0 to 10 min after the addition of 5 mg of DPT-HPP hypercrosslinked polymer to aqueous RhB solution, and then completely disappeared after 15 min. This quick adsorption of the DPT-HPP polymer was also confirmed by switching the color of the aqueous RhB solution from red-violet to colorless within 15 min, as shown in Figure 8**D**. Thus, our DPT-HPP hypercrosslinked polymer exhibited a higher efficiency for the elimination of organic RhB from water, and the removal efficiency of RhB using DPT-HPP polymer reached up to 97% within 15 min (Figure 8**D**). These results indicated that the presence of nitrogen atoms in the backbone structure of the DPT-HPP polymer and its higher surface area (1230 m^2^ g^−1^) can enhance the adsorption process and provide a higher dye removal efficiency than that of the TPE-HPP polymer, which does not contain nitrogen atoms and has a lower surface area (922 m^2^ g^−1^).

**Figure 9:**
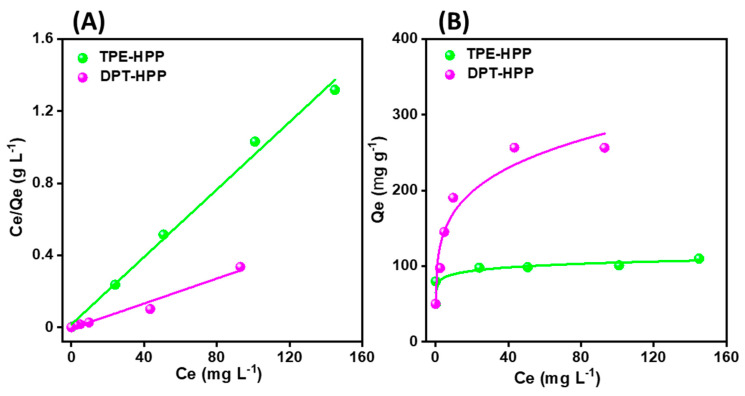
(**A**) Langmuir isothermal models and (**B**) adsorption isothermal curves for the adsorption of the RhB on TPE-HPP and DPT-HPP.

We implemented the Langmuir isothermal model [69] to investigate the kinetic adsorption behavior of the dye RhB on the surface of hypercrosslinked polymers TPE-HPP and DPT-HPP. By fitting the equilibrium adsorption data of our synthesized polymers, the resultant Langmuir isothermal models revealed a linear relationship (C_e_/Q_e_ vs. C_e_) for both TPE-HPP and DPT-HPP polymers (Figure 9**A**). As summarized in Table 2, the correlation coefficients (*R*_L_^2^) of these Langmuir models were 0.99266 for TPE-HPP and 0.96436 for DPT-HPP polymer. According to the resultant Langmuir equations, we calculated the maximum adsorption capacities (*Q*_m_) of our hypercrosslinked polymers, which were found to be 107.41 mg g^–1^ for TPE-HPP and 256.40 mg g^–1^ for DPT-HPP (Figure 9**B**). The *Q*_m_ values confirmed the higher adsorption capacity of DPT-HPP over TPE-HPP. Furthermore, the cyclic adsorption–regeneration test of aqueous RhB dye solution has been utilized to study the reusability of TPE-HPP and DPT-HPP (Figure S7). The adsorption efficiencies of TPE-HPP and DPT-HPP exhibited negligible change after four regeneration cycles, confirming the suitability of our TPE-HPP and DPT-HPP as efficient adsorbents for the removal of RhB from water.

**Figure 10:**
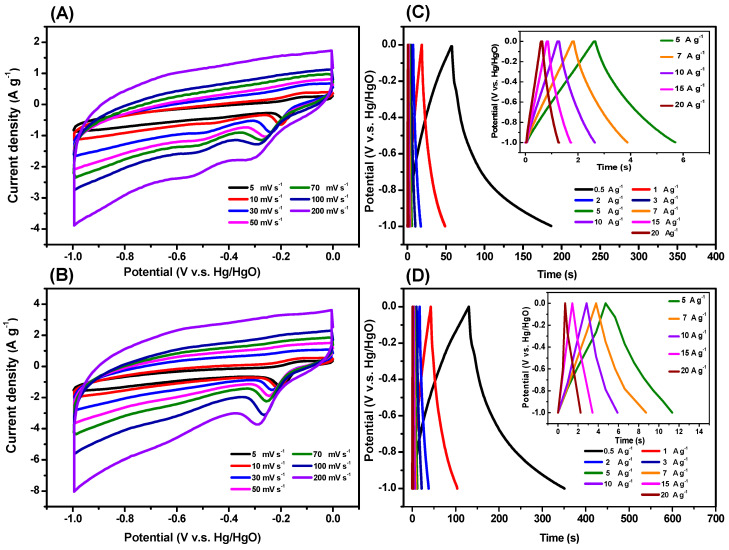
(**A**,**B**) CV and (**C**,**D**) GCD profiles, recorded in 1 M KOH, of the (**A**,**C**) TPE-HPP and (**B**,**D**) DPT-HPP.

To investigate and understand the electrochemical ability of the two porous materials (TPE-HPP and DPT-HPP) in a three-electrode system with 1 M KOH as an aqueous electrolyte, both cyclic voltammetry (CV) and galvanostatic charge-discharge (GCD) were carried out, as shown in Figure 10. The CV curves (Figure 10**A**,**B**) of TPE-HPP and DPT-HPP were recorded in the potential window from 0.00 to –1.00 V (vs. Hg/HgO) with different sweep rates from 5 to 200 mV s^–1^. As shown in their CV profile, both samples displayed humps with rectangle-like shapes, indicating that their capacitive performance is derived from electric double-layer capacitance (EDLC). In addition, the rectangle-like shapes (Figure 10**A**,**B**) of these materials were maintained even at high sweep rates and current densities, confirming the facile kinetics and excellent rate capabilities of TPE-HPP and DPT-HPP. The GCD profiles (Figure 10**C**,**D**) of the TPE-HPP and DPT-HPP revealed that these materials possessed triangular shapes with a slight bend, suggesting a combination of pseudocapacity and EDLC features for these two materials, which arises from the presence of the electroactive methylene groups [46]. In addition, the DPT-HPP sample possessed the redox-active triphenylamine units that can undergo a reversible redox process [30]. As previously reported, in two successive one-electron steps, the tetraphenylethene (TPE) can undergo a redox process, forming its reduced form [70], while the triphenylamine (TPA) possesses a nitrogen atom as its electroactive center connected, to three electron-rich phenyl rings, in which a reversible radical redox process occurs during charge and discharge processes [39,71,72].

**Figure 11:**
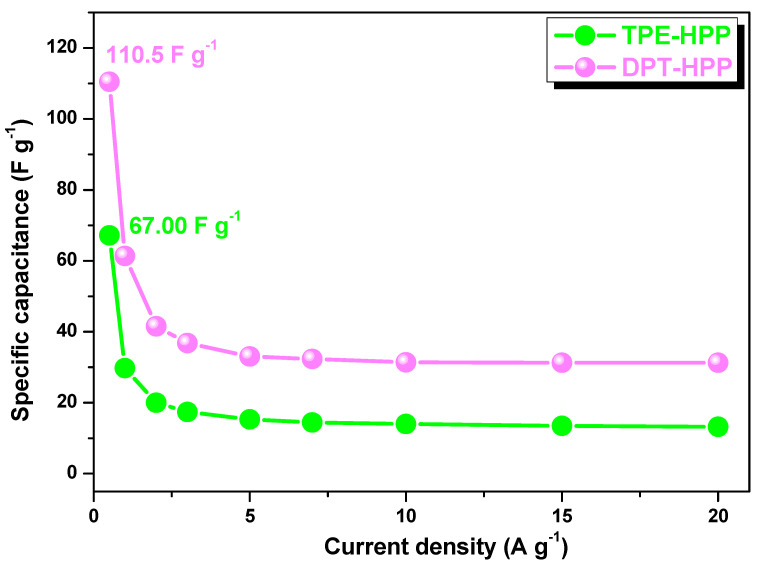
Specific capacitances profile of TPE-HPP and DPT-HPP recorded at various current densities.

As presented in the GCD curves, DPT-HPP had a longer discharging time compared to that of TEP-HPP. Thus, the value of the specific capacitance of the DPT-HPP (110.50 F g^–1^) was higher than that of the TPE-HPP (67.00 F g^–1^), recorded at a current density of 0.5 A g^–1^ (Figure 11). This higher specific capacitance of the DPT-HPP can be attributed to its higher surface area (1230 m^2^ g^−1^), large pore size (2.15 nm), pore volume (1.03 cm^3^ g^−1^) and the fact of its containing heteroatoms such as N atoms in its framework. We found that Car-TPA, Car-TPP, Car-TPT COFs, TPA-COF-1, TPA-COF-2, TPA-COF-3, TPT-COF-4, TPT-COF-5 and TPTCOF-6 exhibited the specific capacitances of 13.6, 14.5, 17.4 F g^–1^, 51.3, 14.4, 5.1, 2.4, 0.34 and 0.24 F g^–1^, respectively, at 0.2 A g^–1^ [30,73]. Khattak and his group revealed that the specific capacitance value of DABTFP COF was 98 F g^–1^ at 0.5 A g^–1^ [74]. Furthermore, the TpPa–COF/PANI composite exhibited a capacitance of 95 F g^–1^ at 0.2 A g^–1^ [75]. In addition, DAAQ–TFP COF showed capacitance of 48 F g^–1^ at 0.1 A g^–1^ [76]. We also reported that An-CPOP-1 and An-CPOP-2 exhibited high specific capacitances of 72.75 and 98.4 F g^–1^, respectively, at 0.5 A g^–1^, and excellent cycling stability [39].

**Figure 12:**
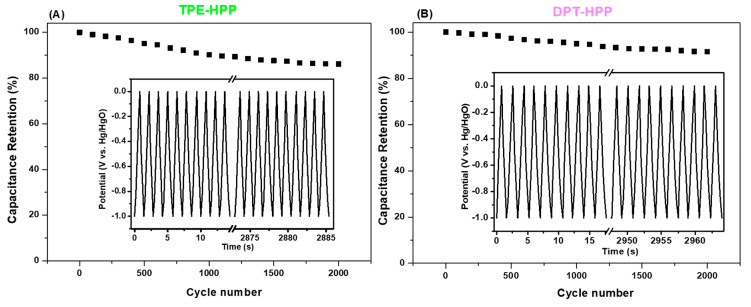
Cycling stability performance of (**A**) TPE-HPP and (**B**) DPT-HPP measured at a current density of 10 A g^−1^ for 2000 cycles.

Table S1 summarizes the specific surface areas and specific capacitances of TPE-HPP, DPT-HPP, other reported POPs, and related materials for supercapacitor applications. Furthermore, the cycling stability of TPE-HPP and DPT-HPP was examined over 2000 repetitions at 10 A g^–1^ (Figure 12), which revealed that TPE-HPP and DPT-HPP had excellent cycling stabilities and capacitance retentions of 86% for TPE-HPP and 92% for DPT-HPP. According to the Ragone plot (Figure S8), these two TPE-HPP and DPT-HPP electrodes showed good energy and power densities.

## 4. Conclusions

According to the FTIR and NMR analyses, two types of HCPs were successfully synthesized through the simple and effective Friedel−Crafts polymerization of tetraphenylethene and 4-(5,6-diphenyl-1H-benzimidazol-2-yl)-triphenylamine with 1,4-bis(chloromethyl)benzene in the presence of FeCl_3_ as a catalyst. Based on dyes and electrochemical measurements, we found that the as-prepared DPT-HPP displayed a significant adsorption capacity (up to 256.40 mg g^–1^) for RhB dye from water, and a supercapacitor performance (up to 110.50 F g^–1^ at a current density of 0.5 A g^–1^) with an excellent cycling stability (up to 95.3% capacitance retention over 2000 cycles). Consequently, our new DPT-HPP can be considered as an excellent candidate for energy storage and dye adsorption applications.

## Figures and Tables

**Table 1:** Thermal stability and porosity properties of TPE-HPP and DPT-HPP, and comparison with their corresponding monomers.

Sample	*T*_d5_ (°C)	*T*_d10_ (°C)	Char Yield (wt.%)	Surface Area (m^2^ g^−1^)	Pore Size (nm)
Ph-2Cl	158	273	62	-	-
TPE	246	263	0	-	-
DPT	186	272	11	-	-
TPE-HPP	387	551	75	922	1.01–1.98
DPT-HPP	263	481	74	1230	1.21–2.15

**Table 2:** Fitted parameters for the adsorption of RhB on the hypercrosslinked polymers TPE-HPP and DPT-HPP.

	*Q*_m_ (mg g^−1^)	*K*_L_	*R*_L_^2^
TPE-HPP	107.41	0.5649	0.99266
DPT-HPP	256.40	0.6250	0.96436

## Supplementary Materials

The following are available online at https://www.mdpi.com/2073-4360/12/10/2426/s1, **Figure S1**: ^1^H NMR spectrum of TPE. **Figure S2**: ^13^C NMR spectrum of TPE. **Figure S3**: ^1^H NMR spectrum of DPT. **Figure S4**: ^13^C NMR spectrum of DPT. **Figure S5**: FT-MS pattern of DPT. **Figure S6**: XRD pattern of (A) TPE-HPP and (B) DPT-HPP. **Figure S7**: Reusability of (A) TPE-HPP and (B) DPT-HPP for the removal of RhB MB within 60 min. **Figure S8**: Ragone plots of the energy density and power density of the (A) TPE-HPP and (B) DPT-HPP electrodes in 1 M KOH. **Table S1**: Comparison between the specific surface area/specific capacitance of TPE-HPP and DPT-HPP with those of previously reported POPs and related materials for supercapacitor applications.
