# Supplementary material for: Multifunctional Hypercrosslinked Porous Organic Polymers Based on Tetraphenylethene and Triphenylamine Derivatives for High-Performance Dye Adsorption and Supercapacitor
Source: Polymers (Basel). 2020 Oct 21;12(10):2426. doi: 10.3390/polym12102426 (PMC7589367; doi:10.3390/polym12102426)
Supplement: Supplementary file 1 [file polymers-12-02426-s001.pdf]

polymers

MDPI

Supporting Information for

Multifunctional Hypercrosslinked Porous Organic Polymers Based on Tetraphenylethene and Triphenylamine Derivatives for High-Performance Dye Adsorption and Supercapacitor

Mohamed Gamal Mohamed [1,2], Ahmed. F. M. EL-Mahdy [1], Tso-Shiuan Meng [1], Maha Mohamed Samy [1] and Shiao-Wei Kuo [1,3,*]

1. Department of Materials and Optoelectronic Science, Center of Crystal Research, National Sun Yat-Sen University, Kaohsiung 80424, Taiwan; mgamal.eldin34@gmail.com (M.G.M.); ahmed1932005@gmail.com (A.F.M.E.-M.); s199483226@gmail.com (T.-S.M.); maha35430@gmail.com (M.S.M.)
2. Chemistry Department, Faculty of Science, Assiut University, Assiut 71516, Egypt
3. Department of Medicinal and Applied Chemistry, Kaohsiung Medical University, Kaohsiung 807, Taiwan

* Correspondence: kuosw@faculty.nsysu.edu.tw; Tel.: +886-7-525-4099

Characterization

FTIR spectra were recorded using a Bruker Tensor 27 FTIR spectrophotometer and the conventional KBr disk method; 32 scans were collected at a spectral resolution of 4 cm^{-1}. The films used in this study were sufficiently thin to obey the Beer-Lambert law. Wide-Angle X-ray diffraction (WAXD) pattern was obscured from the wiggler beamline BL17A1 of the National Synchrotron Radiation Research Center (NSRRC), Taiwan. A triangular bent Si (111) single crystal was used to obtain a monochromated beam having a wavelength (λ) of 1.33 Å. Cross-polarization with MAS (CPMAS) was used to acquire ^{13}C NMR spectral data at 75.5 MHz. The CP contact time was 2 ms; 1H decoupling was applied during data acquisition. The decoupling frequency corresponded to 32 kHz. The MAS sample spinning rate was 10 kHz. Transmission electron microscope (TEM) images were obtained with a JEOL JEM-2010 instrument operated at 200 kV. Field emission scanning electron microscopy (FE-SEM) was conducted using a JEOL JSM7610F scanning electron microscope. Samples were treated via Pt sputtering for 100 s before observation. BET surface area and porosimetry measurements of the prepared samples (ca. 40-100 mg) were performed using a BEL Master™/BEL sim™); ver.3. 0. 0). Nitrogen isotherms were generated through incremental exposure to ultrahigh-purity N_2 (up to ca. 1 atm) in a liquid nitrogen (77 K) bath. Surface parameters were determined using BET adsorption models in the instrument's software (P/P_0 from 0.05 to 0.35). TGA was performed using a TA Q-50 analyzer under a flow of N_2 atmosphere. The samples were sealed in a Pt cell and heated from 40 to 800 °C at a heating rate of 20 °C min^{-1} under a flow of N_2 atmosphere at a flow rate of 60 mL min^{-1}. UV–Vis spectra were recorded at 25 °C using a Jasco V-570 spectrometer, with deionized water as the solvent.

Dye adsorption experiments

Rhodamine B dye was selected to study the efficiency of the hypercrosslinked polymers TPE-HPP and DPT-HPP to remove dye from water. In a typical experiment, polymer (5 mg) was added to an aqueous solution of the dye (RhB, 10 mL) in a glass vial and then the mixture was stirred (for 0, 15, 30, 60, or 90 min (TPE-HPP) and 0, 5, 10, 15, 30, 60, or 90 min (DPT-HPP). The supernatant was then isolated through centrifugation (6000 rpm, 10 min). The UV–Vis spectrum of the isolated supernatant was measured. To obtain adsorption isothermal curves, various concentrations of the aqueous dye (from 12.5 to 150 mg L^{-1}) were used. For each test, the TPE-HPP and DPT-HPP polymer

(5 mg) was added to an aqueous solution of the dye (10 mL) in a glass vial and then the mixture was stirred for 24 h. The supernatant was isolated through centrifugation and its UV–Vis spectrum recorded, to construct the isothermal curve. The adsorption isothermals of RhB was fitted using the Langmuir isothermal model (linear form), expressed as follows:

$$\frac{C_e}{Q_e} = \frac{1}{K_L Q_m} + \frac{C_e}{Q_m} \quad (1)$$

where C_e (mg L^{-1}) is the equilibrium dye concentration in the liquid phase; Q_e (mg g^{-1}) is the equilibrium adsorption of dye per unit mass of the adsorbent carbon; Q_m (mg g^{-1}) is the maximum equilibrium adsorption of dye per unit mass of the adsorbent carbon; and $\mathbf{K}_L$ (L mg^{-1}) is the Langmuir constant. Adsorption reusability tests were performed by adding the TPE-HPP and DPT-HPP polymer (4 mg) to an aqueous solution of the dye (25 mg L^{-1}; 10 mL) and then stirring for 1 h. The supernatant was isolated and its UV–Vis spectrum recorded. The polymer was washed several times with H_2O, EtOH, THF, and acetone to remove the adsorbed dye. After drying the materials overnight at 100 °C, it was used in the next dye removal test.

Electrochemical Analysis

Working Electrode Cleaning: Before use, the glassy carbon electrode (GCE) was polished several times with 0.05-μm alumina powder, washed with EtOH after each polishing step, cleaned via sonication (5 min) in a water bath, washed with EtOH, and then dried in air.

Electrochemical Characterization: The electrochemical experiments were performed in a three-electrode cell using an Autolab potentiostat (PGSTAT204) and 1 M KOH as the aqueous electrolyte. The GCE was used as the working electrode (diameter: 5.61 mm; 0.2475 cm^2). A Pt wire was used as the counter electrode; Hg/HgO (RE-1B, BAS) was used as the reference electrode. All reported potentials refer to the Hg/HgO potential. The GCE was modified with POP slurries, as described elsewhere. The slurries were prepared by dispersing the TPE-HPP or DPT-HPP (45 wt. %), carbon black (45 wt. %), and Nafion (10 wt. %) in EtOH (1 mL) and then sonicating for 1 h. A portion of this slurry (10 μL) was pipetted onto the tip of the electrode, which was then dried in air for 30 min before use. The electrochemical performance was studied through CV at various sweep rates (from 5 to 200 mV s^{-1}) and through the GCD method in the potential range from +0.5 to −1.00 V vs. Hg/HgO at various current densities (from 0.5 to 20 A g^{-1}) in 1 M KOH as the aqueous electrolyte solution.

The specific capacitance was calculated from the GCD data using the following equation [S1-S3]:

$$C_s = (I\Delta t)/(m\Delta V) \quad \text{(S1)}$$

where C_s (F g^{-1}) is the specific capacitance of the supercapacitor, I (A) is the discharge current, ΔV (V) is the potential window, Δt (s) is the discharge time, and m (g) is the mass of the POP on the electrode. The energy density (E, W h kg^{-1}), and the power density (P, W kg^{-1}) were calculated using the following equations:

$$E = 1000C(\Delta V)^2/(2*3600) \quad \text{(S2)}$$

$$P = E/(t/3600) \quad \text{(S3)}$$

Table 1. Comparison between the specific surface area/specific capacitance of TPE-HPP and DPT-HPP with those of previously reported POPs and related materials for supercapacitor applications.

POPs materials	S_{BET} (m^2 g^{-1})	Capacitance	Ref.
Car–TPA COF	1334	13.6 F g^{-1} at 0.2 A g^{-1}	S1
Car–TPP COF	743	14.5 F g^{-1} at 0.2 A g^{-1}	S1
Car–TPT COF	721	17.4 F g^{-1} at 0.2 A g^{-1}	S1
DAAQ–TFP COF	1280	48 ±10 F g^{-1} at 0.1 A g^{-1}	S2
TPA–COF–1	714	51.3 F g^{-1} at 0.2 A g^{-1}	S3
TPA–COF–2	478	14.4 F g^{-1} at 0.2 A g^{-1}	S3
TPA–COF–3	557	5.1 F g^{-1} at 0.2 A g^{-1}	S3
TPT–COF–4	1132	2.4 F g^{-1} at 0.2 A g^{-1}	S3
TPT–COF–5	1747	0.34 F g^{-1} at 0.2 A g^{-1}	S4
TPT–COF–6	1535	0.24 F g^{-1} at 0.2 A g^{-1}	S3
DAB–TFP COF	385	98 F g^{-1} at 0.5 A g^{-1}	S4
HOMCNSs	502	72.79 F g^{-1} at 0.5 A g^{-1}	S5
TpPa–COF/PANI	574	95 F g^{-1} at 0.2 A g^{-1}	S6
An-CPOP-1	700	72.75 F g^{-1} at 0.5 A g^{-1}	S7
An-CPOP-2	1136	98.40 F g^{-1} at 0.5 A g^{-1}	S7
TPE-HPP	922	67.00 F g^{-1} at 0.5 A g^{-1}	This work
DPT-HPP	1230	110.5 F g^{-1} at 0.5 A g^{-1}	This work

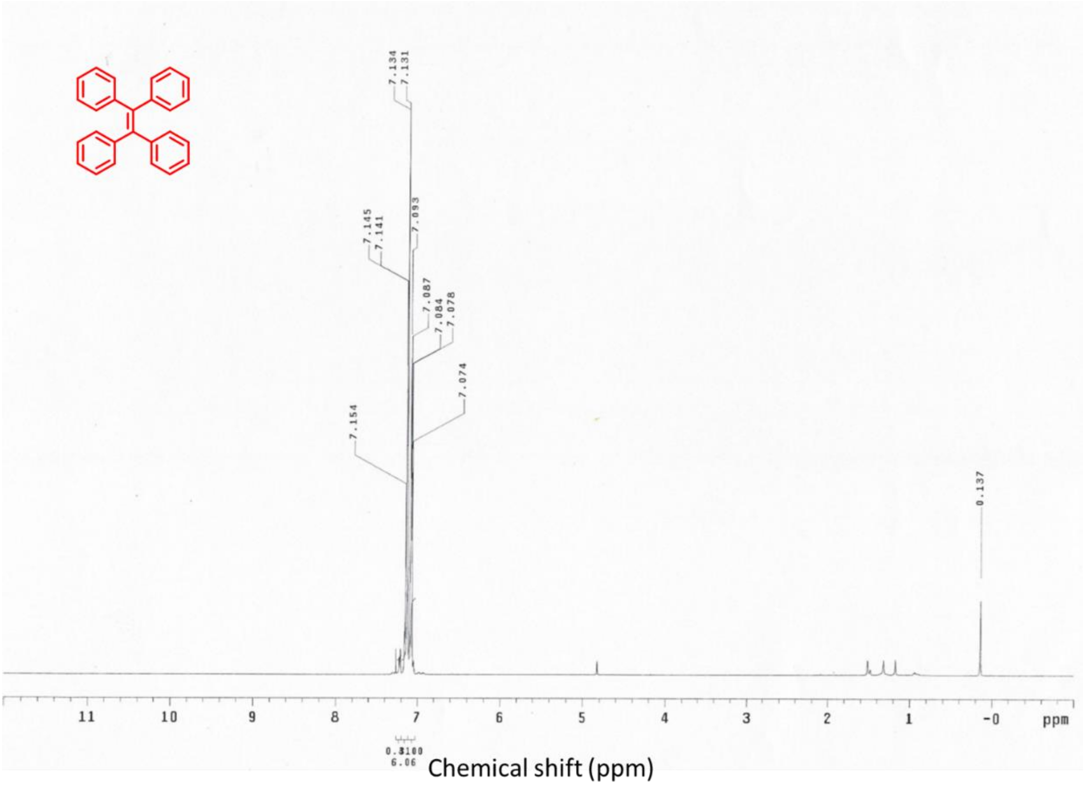

Figure S1. ^{1}H NMR spectrum of TPE.

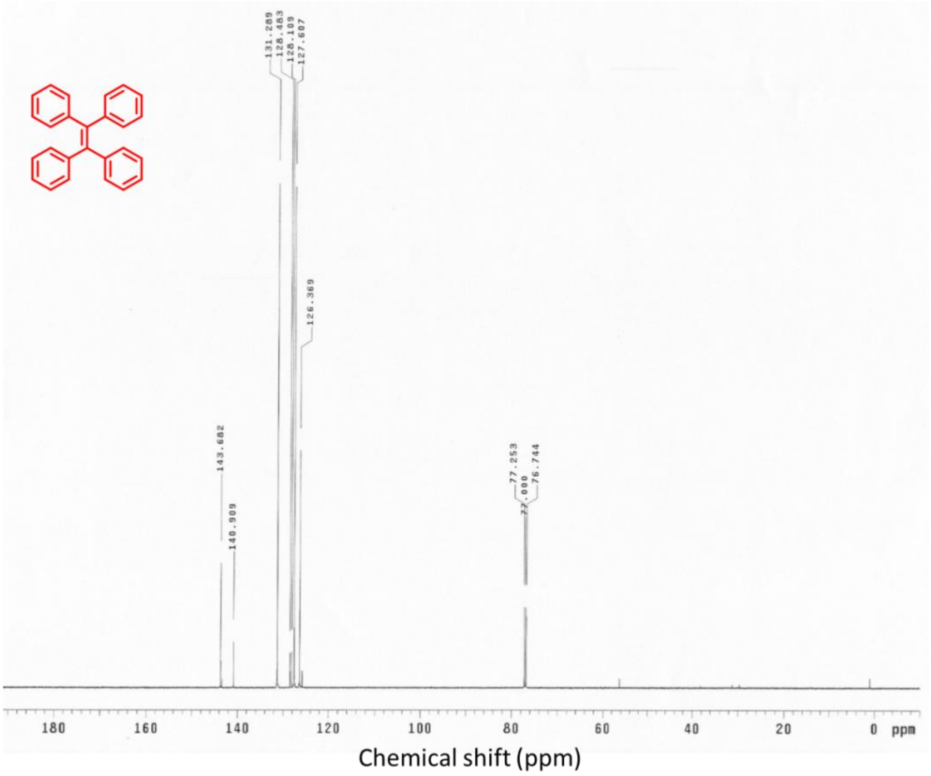

Figure S2. ^{13}C NMR spectrum of TPE.

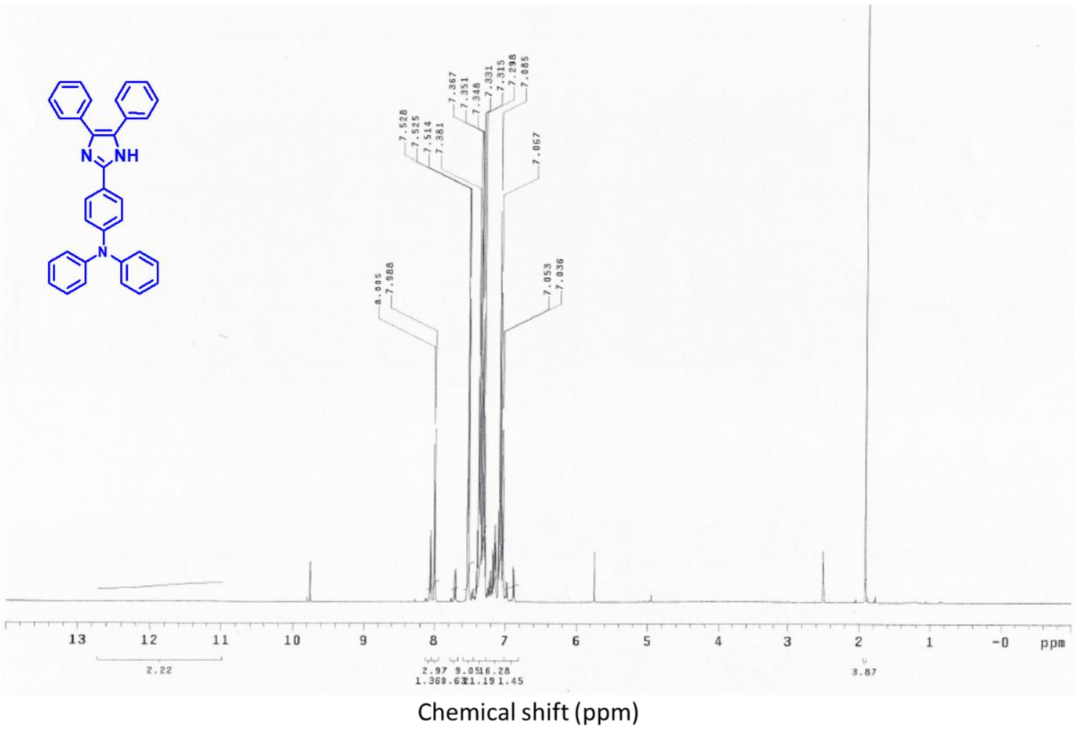

Figure S3. ^{1}H NMR spectrum of DPT.

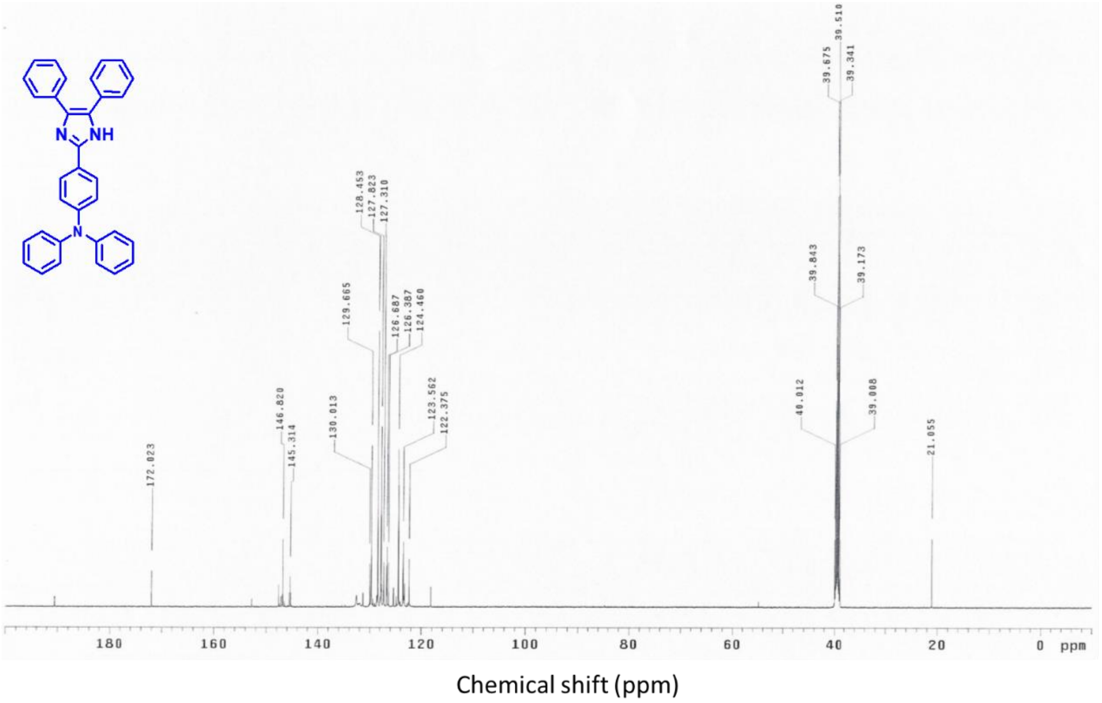

Figure S4. ^{13}C NMR spectrum of DPT.

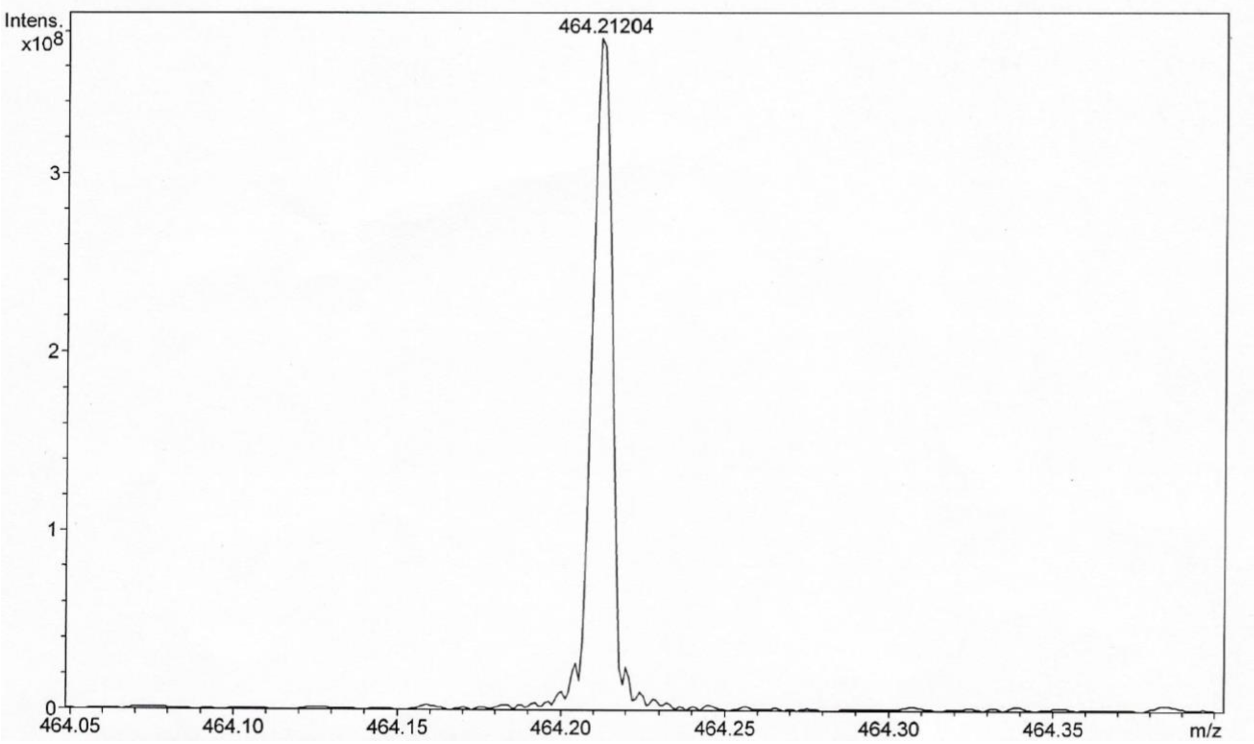

Figure S5. FT-MS pattern of DPT.

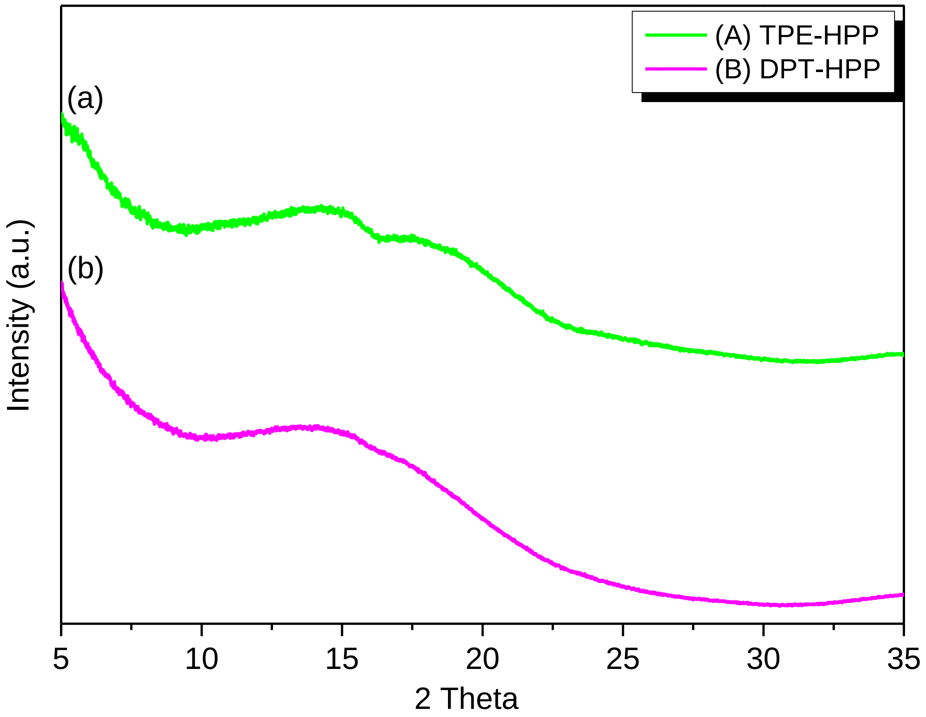

Figure S6. XRD pattern of (A) TPE-HPP and (B) DPT-HPP.

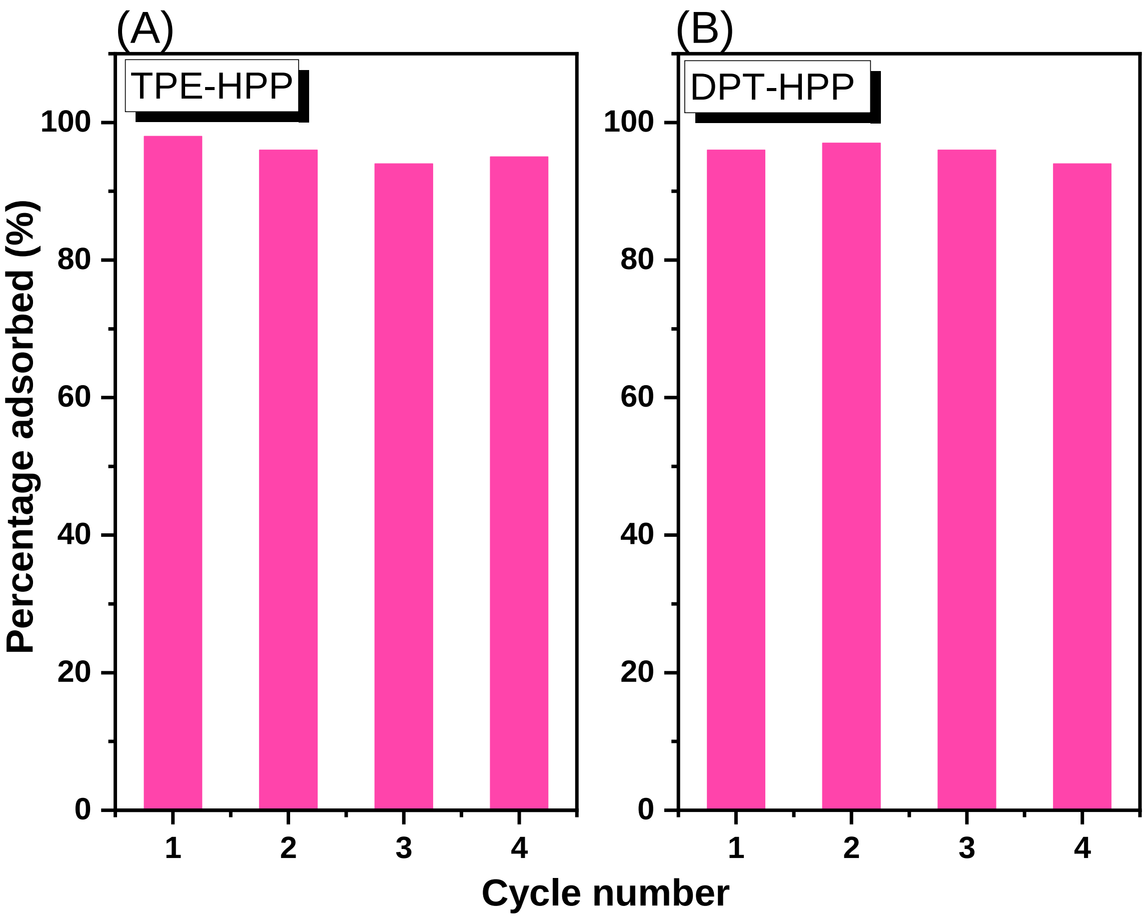

Figure S7. Reusability of (A) TPE-HPP and (B) DPT-HPP for the removal of RhB MB within 60 min.

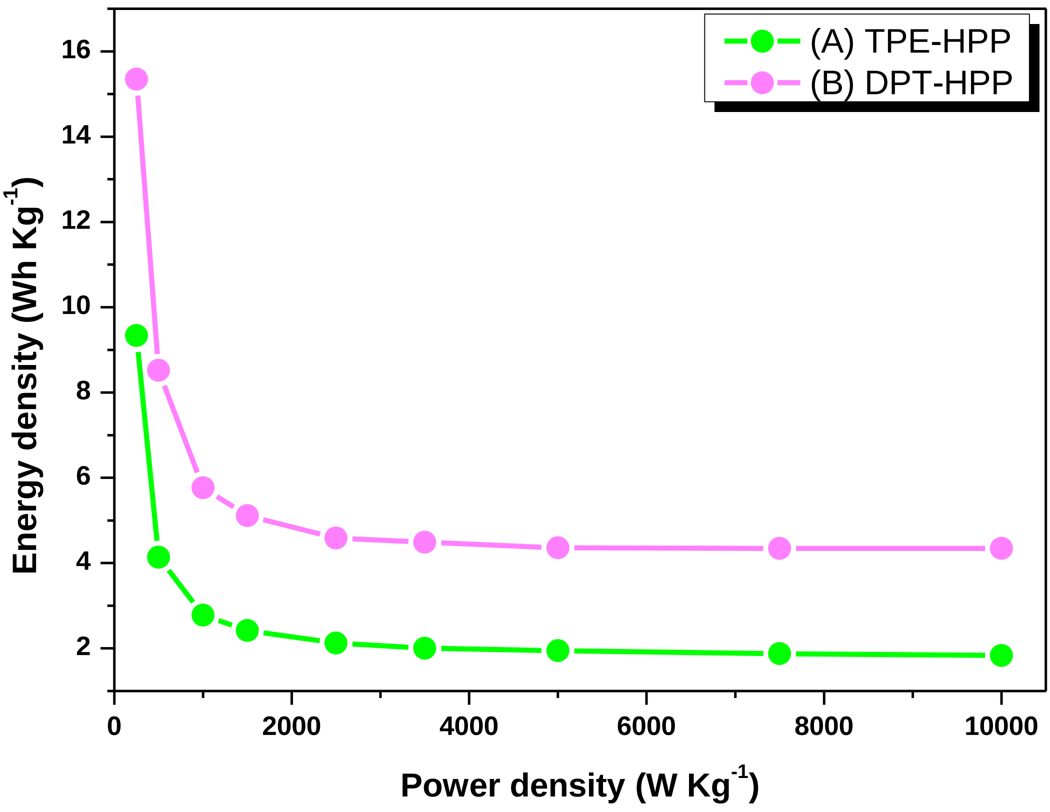

Figure S8. Ragone plots of the energy density and power density of the (A) TPE-HPP and (B) DPT-HPP electrodes in 1 M KOH.

References

[S1] EL-Mahdy, A.F.M.; Young, C.; Kim, J.; You, J.; Yamauchi, Y.; Kuo, S.W.; Hollow Microspherical and Microtubular [3 + 3] Carbazole-Based Covalent Organic Frameworks and Their Gas and Energy Storage Applications, *ACS Appl. Mater. Interfaces*, **2019**, *11*, 9343- 9354.

[S2] DeBlase, C.R.; Silberstein, K.E.; Truong, T.T.; Abruña, H.D.; Dichtel, W.R.; β-Ketoenamine-Linked Covalent Organic Frameworks Capable of Pseudocapacitive Energy Storage, *J. Am. Chem. Soc.*, **2013**, *135*, 16821-16824.

[S3] El-Mahdy, A.F.M.; Kuo, C.H.; Alshehri, A.A.; Kim, J.; Young, C.; Yamauchi, Y.; Kuo, S.W.; Strategic design of triphenylamine- and triphenyltriazine-based two-dimensional covalent organic frameworks for CO_2 uptake and energy storage, *J. Mater. Chem. A*, **2018**, *6*, 19532-19541.

[S4] Khattak, A.M.; Ghazi, Z.A.; Liang, B.; Khan, N.A.; Iqbal, A.; Li, L.; Tang, Z.; A redox-active 2D covalent organic framework with pyridine moieties capable of faradaic energy storage, *J. Mater. Chem. A*, **2016**, *4*, 16312- 16317.

[S5] Ping, J.; Zhang, W.; Yu, P.; Pang, H.; Zheng, M.; Dong, H.; Hu, H.; Xiao, Y.; Liu, Y.; Liang, Y.; Improved ion-diffusion performance by engineering an ordered mesoporous shell in hollow carbon nanospheres, *Chem. Commun.*, **2020**, *56*, 2467-2470.

[S6] Liu, S.; Yao, L.; Lu, Y.; Hua, X.; Liu, J.; Yang, Z.; Wei, H.; Mai, Y. All-organic covalent organic framework/polyaniline composites as stable electrode for high-performance supercapacitors, *Mater. Lett*. **2019**, *236*, 354-357.

[S7] Mohamed, M.G.; X. Zhang, X.; Mansoure, T.H.; EL-Mahdy, A.F.M.; Huang, C.F.; Danko, M.; Xin, Z.; Kuo, S.W. Hypercrosslinked porous organic polymers based on tetraphenylanthraquinone for CO_2 uptake and high-performance supercapacitor, *Polymer* **2020**, *205*, 122857.
